# Identifying the status of genetic lesions in cancer clinical trial documents using machine learning

**DOI:** 10.1186/1471-2164-13-S8-S21

**Published:** 2012-12-17

**Authors:** Yonghui Wu, Mia A Levy, Christine M Micheel, Paul Yeh, Buzhou Tang, Michael J Cantrell, Stacy M Cooreman, Hua Xu

**Affiliations:** 1 Department of Biomedical Informatics, Vanderbilt University, School of Medicine, 2209 Garland Ave, Nashville, TN 37232, USA; 2 Department of Medicine, Division of Hematology and Oncology, Vanderbilt University, School of Medicine, USA; 3 Vanderbilt-Ingram Cancer Center, Vanderbilt University Medical Center, USA

## Abstract

**Background:**

Many cancer clinical trials now specify the particular status of a genetic lesion in a patient's tumor in the inclusion or exclusion criteria for trial enrollment. To facilitate search and identification of gene-associated clinical trials by potential participants and clinicians, it is important to develop automated methods to identify genetic information from narrative trial documents.

**Methods:**

We developed a two-stage classification method to identify genes and genetic lesion statuses in clinical trial documents extracted from the National Cancer Institute's (NCI's) Physician Data Query (PDQ) cancer clinical trial database. The method consists of two steps: 1) to distinguish gene entities from non-gene entities such as English words; and 2) to determine whether and which genetic lesion status is associated with an identified gene entity. We developed and evaluated the performance of the method using a manually annotated data set containing 1,143 instances of the eight most frequently mentioned genes in cancer clinical trials. In addition, we applied the classifier to a real-world task of cancer trial annotation and evaluated its performance using a larger sample size (4,013 instances from 249 distinct human gene symbols detected from 250 trials).

**Results:**

Our evaluation using a manually annotated data set showed that the two-stage classifier outperformed the single-stage classifier and achieved the best average accuracy of 83.7% for the eight most frequently mentioned genes when optimized feature sets were used. It also showed better generalizability when we applied the two-stage classifier trained on one set of genes to another independent gene. When a gene-neutral, two-stage classifier was applied to the real-world task of cancer trial annotation, it achieved a highest accuracy of 89.8%, demonstrating the feasibility of developing a gene-neutral classifier for this task.

**Conclusions:**

We presented a machine learning-based approach to detect gene entities and the genetic lesion statuses from clinical trial documents and demonstrated its use in cancer trial annotation. Such methods would be valuable for building information retrieval tools targeting gene-associated clinical trials.

## Background

In recent years, novel cancer therapies targeting specific genetic lesions in tumors have shown great promise for improving outcomes for patients with cancer [1,2]. Basic and clinical research has revealed that some genetic lesions are not only necessary for the initial development or progression of a specific tumor but are also required for the maintenance of that tumor's survival; a concept referred to as 'oncogene addiction' [3]. Many clinical trials for targeted therapies now specify the particular status of a genetic lesion as detected or not detected in the inclusion or exclusion criteria for trial enrolment. Genetic lesions in tumors may be detected as an abnormal gene mutation, gene rearrangement, gene amplification, or gene-product (protein) expression. However, clinical trial databases such as ClinicalTrials.gov (http://www.clinicaltrials.gov) and the National Cancer Institute's Physician Data Query (PDQ) database [4], do not contain structured representations of clinical trial eligibility criteria, making it difficult to accurately search for trials based on the status of the genetic lesion.

MyCancerGenome.org, developed and hosted at Vanderbilt University, is a freely available online knowledge base that summarizes the clinical significance of specific tumor genetic lesions for given cancer diagnoses. Users are informed about standard of care therapies and gene-associated clinical trials open to accrual locally, nationally, and internationally. At MyCancerGenome.org, users can search for clinical trials by gene and disease. The current implementation of the search function uses a simple keyword search against all PDQ clinical trial documents in the database. However, exact string matching of gene symbols alone often leads to false positive results due to the ambiguity of gene symbols. For example, searching gene symbol "MET" could return clinical trial documents containing the English word "met" (e.g., "patient has *met *the inclusion criteria ..."). In addition, gene symbols could be mentioned as part of other biomedical entities. In the sentence "patients may not have had prior EGFR Tyrosine Kinase Inhibitors", the term "EGFR" refers not to the gene *EGFR *but rather to the drug class "EGFR Tyrosine Kinase Inhibitors". Furthermore, the status of a genetic lesion as detected or not detected is often included in clinical trial eligibility criteria. For example, a clinical trial may specify an inclusion criterion "with a positive mutational status for the BRAF or MEK1 gene." Ideally, we would like to allow a clinician or patient to filter the list of potential clinical trials based on the status of an individual's particular genetic lesion. Therefore, it is essential to identify the status of genetic lesions in clinical trial documents. The simple keyword search method cannot meet these requirements. In this study, we thus sought to use more advanced text processing methods to distinguish gene symbols from English words and other biomedical entities, as well as to determine the status of genetic lesions in clinical trial documents.

Identifying gene instances and genetic lesion statuses can be viewed as a particular case of a word sense disambiguation (WSD) problem, which is a classification task involving assignment of the correct sense of a particular occurrence of an ambiguous term. In biomedical literature, ambiguity of gene names is significant [5]. An ambiguous gene symbol could refer to: 1) multiple genes; 2) a gene or an English word not related to a gene; 3) an RNA, protein, or gene; or 4) genes in different species. Many studies have been done to recognize and normalize gene names in biomedical literature. One example is BioCreAtivE, tasks I and II in particular [6-8], which were designed to address those problems; most groups participating in the tasks achieved F-scores between 0.75 and 0.85 [9-12]. Some studies focused on disambiguation of gene symbols: for example, among different semantic types or different genes. Hatzivassiloglou et al. [13] conducted a study to disambiguate gene names among three semantic classes: gene, RNA, and protein, and reported accuracy rates up to 85% with optimized feature combinations. Podowski et al. [14] built a two-step classification system (the first classifier for AllGenes versus NotGene, followed by the second classifier for determining the appropriate gene) and they reported an F-measure of over 0.7 for >7,000 genes with sufficient number of known document references. Furthermore, a number of studies investigated different methods for disambiguation among different gene senses and showed great performance (with precision over 95%) [15-17]. More recently, a study by Stevenson and Guo [18] also demonstrated that multiple features and methods would be required for optimal resolution of different types of gene symbol ambiguity in biomedical literature.

Almost all previous work on gene symbol disambiguation has focused on the biomedical literature. Little attention has been paid to clinical trial documents that increasingly include eligibility criteria referring to patient genetic information. In this study, we developed a new two-stage classifier to identify gene entities and associated status of genetic lesions from clinical trial documents. The classifier identifies gene entities first and then determines genetic lesion status. Our evaluation using a manually annotated data set containing instances of the top eight most frequently mentioned genes in cancer clinical trials showed that the classifier with optimized feature sets achieved a best average accuracy of 83.7%. When it was applied to a real-world task of annotating mentions of any human gene in cancer trials, the two-stage classifier achieved a highest accuracy of 89.8%. To the best of our knowledge, this is the first attempt to determine the status of genetic lesions in clinical trial documents.

## Methods

### Overview

In this study, we selected the top eight most frequently mentioned gene symbols in the NCI's PDQ cancer clinical trials database. For each gene symbol and its synonyms, up to 200 occurrences of the gene symbol were randomly selected and reviewed by domain experts to assign one of the six predefined status categories. Using the annotated data set for each gene, we developed and evaluated gene-specific classifiers for gene entity and genetic lesion status, using the Support Vector Machines (SVM) algorithm. In addition, we assessed the feasibility of building a general, gene-neutral classifier that can apply to any gene. We then evaluated the gene-neutral classifier first using samples of the top eight most frequently mentioned cancer genes and then using instances of all HGNC human genes detected from 250 randomly selected cancer trials.

### Data sets

The NCI's PDQ cancer database contains descriptions of cancer clinical trials conducted around the world dating back to the 1970's. PDQ is freely available for download in XML format with weekly updates [4]. For this study, we used the collection of PDQ clinical trials downloaded on February 6, 2012. This data set contained descriptions for over 11,443 active clinical trials of which we used a subset of 6,949 therapeutic trials, and 14,926 closed clinical trials of which we used a subset of 13,790 therapeutic trials. For this study, we used a subset of the trial description sections including trial title, summary, and eligibility criteria. The closed clinical trials in PDQ were used as a development set, where our developers could look into those unannotated data. The active trials in PDQ were used for training and testing of the classifier--samples were randomly selected and manually reviewed by domain experts to build annotated data sets, as discussed in the next paragraph.

We used a list of 33,128 approved human genes from the HGNC (HUGO Gene Nomenclature Committee)[19] database, of which 446 genes have been classified as cancer genes in the Catalogue of Somatic Mutations in Cancer (COSMIC) database [20]. Each gene was associated with a set of synonyms obtained from the HGNC database and the National Center for Biotechnology Information's Gene database (NCBI Gene) [21]. We searched for the 33,128 HGNC human genes in the 6,949 active therapeutic PDQ clinical trials and ranked them by frequency. The top eight most frequently mentioned cancer gene symbols from the list of cancer genes in the COSMIC database were used in the first part of this study: ALK, BRAF, EGFR, KIT, KRAS, MET, PTEN and WT1. For each gene symbol, we collected all occurrences in PDQ trial documents based on simple string matching. We then randomly selected 200 occurrences (or the maximum number of gene occurrences if less than 200) for each gene and sent them to two domain experts for independent annotation. The annotator read the context (a sentence or relevant sections if needed) where a gene symbol occurred and assigned one of six predefined status categories to the gene mention. Table 1 shows the definitions and examples of the six categories, which were defined based on manual observation and needs for searching clinical trials. Categories 5 and 6 are not gene-related and categories 1-4 refer to the status of a genetic lesion. To facilitate the annotation, we also developed annotation guidelines that provided definitions and examples for each category. Each annotator independently annotated all samples; we have reported the inter-annotator agreement (IAA) using the Kappa score.

**Table 1:** Six categories of gene mentions in clinical trial documents.

Category			Definition	Examples
**ID**	**Stage I**	**Stage II**		

1	Gene-related	Genetic lesion detected	Genetic lesion status is detected.	• Positive EGFR mutation test... • Patient with EGFR positive ...
		
2		Genetic lesion not detected	Genetic lesion status is Not Detected.	• ...negative staining for Kit. • Patient must have wild type KRAS.
		
3		Genetic lesion mentioned	Analysis of genetic lesion is mentioned but not particular results	• BRAF - gene analysis of archival tissue • mutational analysis of genes such as EGFR ...
		
4		Gene only	It refers to the gene entity only, no status is associated.	• KIT is a gene that codes for ... • WT1 is a protein in cancer cells that regulates gene expression and ...

5	Drug		Gene related drugs, drug classes, or other therapy	• WT1 Peptide Vaccination in Carcinomas. • Prior treatment with EGFR inhibitor chemotherapy...

6	Others		None of the above classes, e.g., English words,	• ...using the kit and testing procedures. • Criteria are met.

After individual annotation, we collected all the discrepancies between the two annotators' results and presented them to the annotators. They manually reviewed those discrepancies and made their final designations, which led to our gold standard.

### Feature sets

Based on previous studies in named entity recognition and disambiguation in biomedical text [16], we investigated four feature categories for the classification tasks in this study:

Contextual words and associated information within a window: a) words within a window size of 6 for a target gene symbol; b) direction (e.g., left or right) of the feature words; c) distance of the feature words; and d) orthographic information of the gene symbol and its closest words (e.g., if a word contains capital letters, digits, or special characters).

Words with dependency relationships to the gene symbol. We used the Stanford Parser [22] to generate a dependency parse tree for each sentence containing the gene symbol. From the parse tree, we identified words with a dependency relationship to the gene symbol. Then we used those words, their part of speech (POS) tags, and the type of dependency relationship as features. For example, for the phrase "pRCC histology with Y1230 or D1228 MET mutations", the Stanford Parser would generate a dependency relationship "amod (mutations, MET)", denoting that "MET" is an adjectival modifier of "mutations". Therefore, "mutations", its POS tag, and the dependency relationship "amod" will be used as features in this example.

Words expressing negation status. We noticed that "Genetic lesion not detected" status was often associated with a list of common negation words, which should be a valuable feature. We manually reviewed about 200 documents from the closed trials in PDQ and complied a list of negation words. Whether there are negation words in a window size of 3 of the target gene symbol was then used as a feature for the classifier.

Section headers. Three section headers, including "Title", "Summary" and "Eligibility criteria" were used in this study.

### Gene-specific classifiers

As we have an annotated data set for each of the top eight genes, we first tested gene-specific classifiers for this task. We built eight classification systems--one for each gene--and evaluated them using annotated samples.

As shown in Table 1, there is a hierarchical structure among six categories--categories 1-4 can be viewed as sub-classes of a new pseudo-category "Gene-related", which is at the same level as categories 5 (Drug) and 6 (Others). Therefore, we proposed a new two-stage classification system for this task. Stage 1 was to classify among "Gene-related" (merging categories 1-4), "Drug", and "Others". In stage 2, we built a classifier to further divide the "Gene-related" class into four categories of genetic lesion status. For both steps, SVM was used as the machine learning algorithm because of its known high performance. The LibSVM package [23] was used in this study. In addition, a single-stage SVM classifier, which classifies among all six categories in one step, was also implemented; this served as the baseline method in the study.

As discussed in the previous paragraph, for each of the top eight gene symbols, we built a two-stage classifier and a single-stage classifier based on annotated samples of that gene symbol. The classifiers were developed and evaluated using 5-fold cross validation. We optimized combinations of features and parameters of SVM based on the average accuracy of five test folds in the cross validation.

### Gene-neutral classifiers

As described above, we built a classifier for each individual gene, which required creating an annotated data set for each gene. Because manual annotation is costly and time-consuming, this approach lacks scalability, especially when more and more genes are associated with diseases and the number of gene-associated clinical trials continues to increase. Therefore, it is in our interest to investigate the possibility of building a general classifier that can apply to new genes, by training on samples from a limited set of genes. In this study, we tested the feasibility of such a gene-neutral classifier. For each of the top eight genes, we merged all annotated samples from the other seven genes and used them to train a classifier, and then we applied the classifier to samples of the selected gene and reported its performance. Basically, we trained a classifier using seven genes and tested it on the eighth gene, and repeated this process eight times. Finally, we compared the performance of the gene-neutral classifiers with individual gene-specific classifiers (see Tables 3 and 5).

In addition, we applied the gene-neutral classifier to the real-world task of cancer trial annotation of mentions of any gene in the HGNC list of human genes (33,128 in total). To analyze the correlation between the system's performance and sample size, we increased the number of annotated trials and reported the classifier's performance in an iterative approach. We started with the classifier trained on annotated samples from the top eight genes and then retrained and retested the gene-neutral classifier over five iterations. For each iteration, fifty cancer trial documents were randomly selected, and the gene names and synonyms mentioned in these trials were identified by a string-matching program. The gene-neutral classifier was then used to predict the status of each gene symbol instance. A domain expert manually reviewed and corrected the labels predicted by the classifier; the domain expert annotations served as the gold standard. We reported the performance of the classifier on genes in the 50 trials based on that gold standard. Next, annotated samples from the 50 trials were combined with existing annotated samples and used to build an updated gene-neutral classifier for the next iteration. We repeated this procedure five times and recorded the system's accuracy at each iteration.

### Evaluation

As this was a typical multi-class classification task, we used accuracy as the primary measurement for classification performance. Predicted results from our classifiers were compared to the gold standard to determine if an instance was correctly assigned to a category. Accuracy was defined as the ratio between the number of correctly predicted instances by the system and the number of total instances in a test set. For each individual gene, 5 test folds were be generated from the 5-fold cross validation. Prediction results from all 5 test folds were combined and used to calculate an overall accuracy, which was the final accuracy for that gene, as shown in Table 4. We also reported average accuracy across the top eight genes.

To further analyze classifier performance in each category, we reported the precision, recall, and F-score for individual categories. As an individual gene may have limited instances for a particular category, we merged classification results from the top eight genes and reported category-specific performance on the merged data set. The precision of a category was defined as the ratio between the number of instances that were correctly predicted to belong to that category and the number of all instances predicted to belong to that category. The recall for a category was defined as the ratio between the number of instances that were correctly predicted to belong to that category and the number of all instances in the category based on the gold standard. The F-score for the category was then calculated as: 2 * Precision * Recall/(Precision + Recall).

## Results

### Characteristics of the data set and annotation result

**Figure 1:**
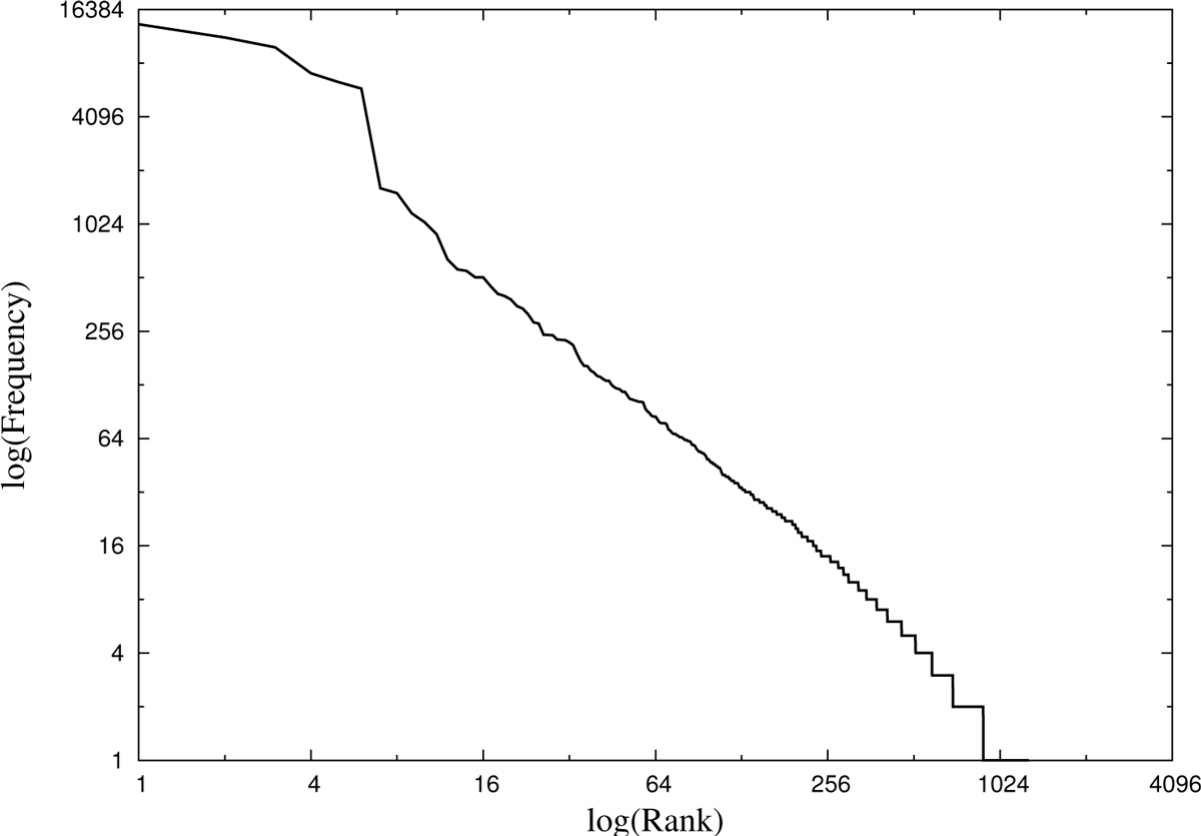
**Frequency distribution of detected gene symbols detected in cancer trial documents**.

Using the list of all human genes from the HGNC database, 1,290 individual gene symbols were detected in 4,951 trials. Figure 1 shows the frequency distribution for all the detected gene symbols, which were ranked by the number of occurrences.

Table 2 shows the number of instances in each category for the top eight cancer gene symbols, based on manual annotation. As we can see, the frequency distribution among categories was very different among the top eight gene symbols. Genes "EGFR" and "MET" were highly unbalanced, with a major sense of **"**Drug**" **or **"**Others**"**, respectively. For each gene symbol, there existed categories with only a few instances (less than 5).

**Table 2:** Frequency distribution among different categories for the top eight genes.

Gene	# of Samples	Others	Drug	Genetic lesion detected	Genetic lesion not detected	Genetic lesion mentioned	Gene only
ALK	102	32	10	41	15	4	0

BRAF	130	0	32	63	13	21	1

EGFR	200	4	117	37	5	26	11

KIT	145	5	26	82	12	16	4

KRAS	200	2	0	65	95	38	0

MET	200	147	28	16	0	5	4

PTEN	69	4	2	41	1	19	2

WT1	97	0	53	32	0	9	3

**Total**	1,143	194	268	377	141	138	25

Among all 1,143 instances from the top eight gene symbols, the two annotators annotated 148 instances differently. The calculated Kappa score between two annotators was 0.837, which indicates a reasonable agreement between two annotators. Forty-eight discrepancies were between "Genetic lesion detected" and "Genetic lesion not detected" and twenty-seven were between "Genetic lesion detected" and "Gene only", which suggested that discrimination among gene-related categories was more difficult than that between gene and the non-gene categories "Others" and "Drug".

### Performance of gene-specific classifiers

Table 3 shows accuracies of the two-stage and single-stage classifiers that were trained and tested on each individual gene, when optimized features and parameters were used. For all of the top eight genes, the two-stage SVM classifier always performed better than or equal to the single-stage SVM classifier. The average accuracy across the top eight genes was 83.7% for the two-stage classifier and 83.2% for the single-stage classifier. We further evaluated the performance of the first classifier (for determining "Others", "Drug" and "Gene-related") in the two-stage approach; it achieved an average accuracy of 94.4% across the top eight genes.

**Table 3:** Accuracy of two-stage and single classifiers for individual genes.

Gene	Two-stage classifier	Single-stage classifier
ALK	88.2%	88.1%

BRAF	85.4%	85.4%

EGFR	82.5%	82.0%

KIT	75.9%	75.2%

KRAS	87.5%	87.5%

MET	93.0%	92.5%

PTEN	73.9%	71.3%

WT1	83.5%	83.5%

**Average **	**83.7%**	**83.2%**

Table 4 shows the precision, recall, and F-score for each category using all instances from the top eight genes, for both the two-stage classifier and the single-stage classifier. The six categories showed very different performance. "Others" and "Drug" had higher F-scores, while the "Gene only" category had a low F-score. This probably occurred because the "Gene only" category had the smallest number of training samples (25 total across all genes), and instances appeared in similar contexts as compared to other categories of gene instances. The two-stage classifier showed a better F-score than that of the single-stage classifier for four categories, although it slightly dropped performance for the "Drug" and "Genetic lesion detected" categories.

**Table 4:** Precision, Recall and F-score for individual categories across the top eight genes.

	Two-stage classifier			Single-stage classifier		
**Category**	**Precision**	**Recall**	**F-score**	**Precision**	**Recall**	**F-score**

Genetic lesion detected	75.4%	92.0%	82.9%	78.4%	89.4%	83.5%

Genetic lesion not detected	90.2%	78.0%	83.7%	89.4%	78.0%	83.3%

Genetic lesion mentioned	78.5%	60.9%	68.6%	83.5%	55.1%	66.4%

Gene only	66.7%	16.0%	25.8%	60.0%	12.0%	20.0%

Drug	91.0%	90.3%	90.6%	82.0%	95.2%	88.1%

Others	100%	93.8%	96.8%	100%	94.3%	97.1%

### Performance of gene-neutral classifiers

Table 5 shows the performance of gene-neutral classifiers on the top eight genes using the two-stage and single-stage approaches. As expected, gene-neutral classifiers had lower performance than gene-specific classifiers: the average accuracy dropped from 83.7% (Table 3) to 76.3% (Table 5) for two-stage classifiers. However, the two-stage classification approach showed much better results (average accuracy of 76.3%) than the single-stage classifier (average accuracy of 68.8%), indicating better generalizability across genes. In addition, individual gene symbols performed differently when gene-neutral classifiers were applied. For example, gene "PTEN" had a better accuracy for gene-neutral classifier (78.3%) than that of the gene-specific classifier (73.9%). However, the accuracy of gene "MET" dropped significantly--from 93.0% for the gene-specific classifier to 73.5% for the gene-neutral classifier.

**Table 5:** Accuracy of the gene-neutral two-stage and single classifiers.

Testing Gene	Training Genes	Two-stage Classifier	Single-stage Classifier
ALK	BRAF, EGFR, KIT, KRAS, MET, PTEN, WT1	68.6%	64.7%

BRAF	ALK, EGFR, KIT, KRAS, MET, PTEN, WT1	85.4%	84.6%

EGFR	ALK, BRAF, KIT, KRAS, MET, PTEN, WT1	78.0%	73.5%

KIT	ALK, BRAF, EGFR, KRAS, MET, PTEN, WT1	74.5%	70.3%

KRAS	ALK, BRAF, EGFR, KIT, MET, PTEN, WT1	87.0%	81.5%

MET	ALK, BRAF, EGFR, KIT, KRAS, PTEN, WT1	73.5%	41.5%

PTEN	ALK, BRAF, EGFR, KIT, KRAS, MET, WT1	78.3%	78.3%

WT1	ALK, BRAF, EGFR, KIT, KRAS, MET, PTEN	65.0%	55.7%

**Average**		**76.3%**	**68.8%**

**Figure 2:**
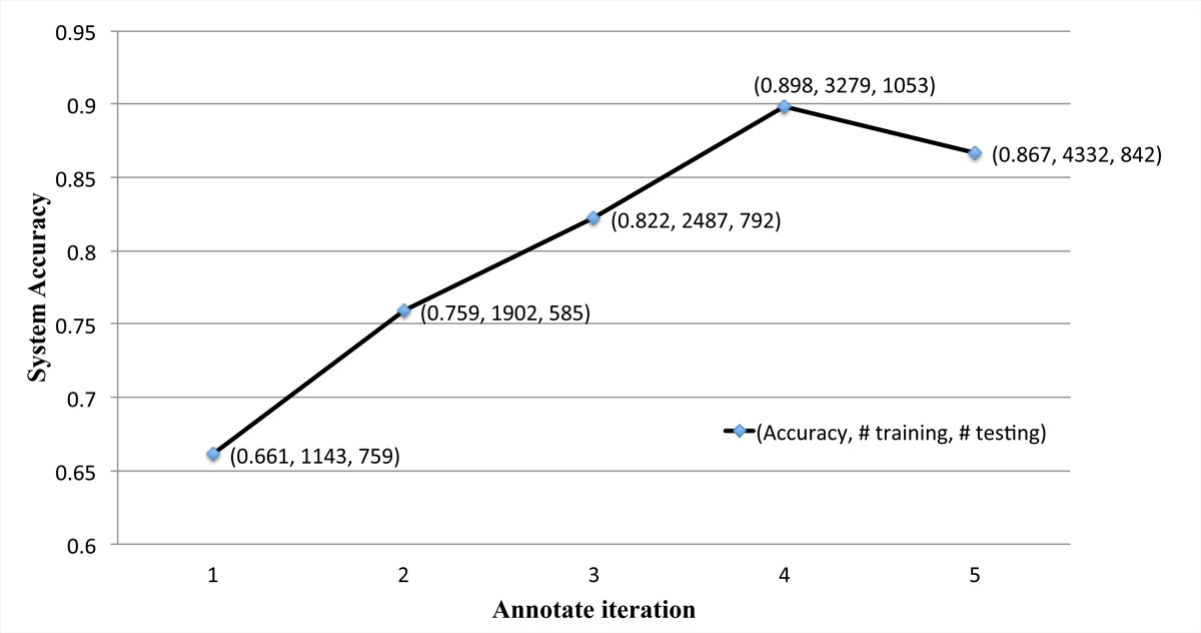
**Performance of the gene-neutral classifier at each iteration**. The triplet values at each point represent the accuracy, the number of training samples, and the number of test samples respectively.

To apply the gene-neutral classifier to the real-world task of annotating mentions of any gene in cancer trial documents, we generated a set of 4,031 annotated gene instances (including 249 distinct gene symbols) from 250 randomly selected trial documents. The numbers of annotated samples over the five iterations were 759, 585, 792, 1053, and 842, respectively. A new status category, named "Genetic lesion detected or not detected", which denotes that a patient would meet eligibility criteria as long as their genetic status had been measured prior to trial entry, was identified and added to the annotation guidelines. Figure 2 shows the system's performance at each iteration point. Due to the new category, we obtained a low accuracy of 66.1% from the first iteration, which was trained on gene instances from the top eight genes. As new training samples were added, the retrained gene-neutral classifier improved its performance. After three iterations (including 3,279 annotated samples), the gene-neutral classifier achieved a highest accuracy of 89.8%. The accuracy after the fifth iteration dropped (probably due to the variability in the fifth set of trial documents), but it still retained a reasonable accuracy of 86.7%.

## Discussion

With the rapid growth in the field of personalized medicine, the rate at which clinical trials are being opened with eligibility criteria referring to patient genetic information is accelerating. To efficiently search those trials, gene-specific information such as genetic lesion status needs to be accurately identified and categorized from narrative clinical trial protocols. As an initial step, we developed a machine learning-based system to identify gene entities and the status of genetic lesions from clinical trial descriptions. The gene-specific classifier achieved an average accuracy of 83.7% for the eight genes included in the first part of this study. When it was applied to all genes in cancer trial documents, the gene-neutral classifier achieved a highest accuracy of 89.8%, indicating the system's potential in facilitating information retrieval tasks targeting clinical trial documents.

In our experiment, the two-stage classifier outperformed the single-stage classifier for both gene-specific and gene-neutral tasks. The two-stage classification idea is related to work published by Podowski et al. [14], where they built an "AllGenes vs. NotGene" classifier followed by a "Gene vs. OtherGene" classifier to identify gene names in biomedical literature. Staged classification strategies may have originated in the work of Bennett and Blue [24], where they built cascade SVM classifiers to generate a decision tree. Our rationale behind using this design was that the classification models might be different between the stage 1 classifier (to determine "Others", "Drug", and "Gene_related") and the stage 2 classifier (to determine different status categories for genetic lesions). After the first classification, there may have been less noise added to the second classification, as non-gene classes were excluded. Results stratified by individual categories in Table 4 also showed that the level of difficulty for each category was different: "Others" and "Drug" categories were relatively easier, while other gene-related categories were more difficult. In addition, analysis of disagreement between two annotators showed that most of discrepancies were also from gene-related categories.

We investigated the contribution of different types of features for this task. For gene-specific classifiers, the highest accuracy (83.2%) was achieved by combining all four categories of features (see Methods). Other combinations achieved lower performance, e.g., 81.4% for using category 1 features, 82.2% for using category 1 and 2 features. Another interesting finding was that direct use of POS tagging information did not improve the performance. But when POS tags of words that have a dependency relationship with the gene symbol were used, the system's performance was improved, which indicates the value of dependency relationships in this task.

The experimental results from the gene-neutral classifiers were very interesting to us. It was not surprising that the gene-neutral classifier had a lower average accuracy across the top eight genes, when compared with the gene-specific classifier. But a few genes had almost no or little loss in performance, including "BRAF", "KIT", and "KRAS". The gene-neutral classifier even had a better performance than gene-specific classifier for gene "PTEN". This could be related to the increased sample size by merging annotated data from other seven genes, as "PTEN" alone had only 69 annotated samples. A couple of genes had big performance losses when we switched from a gene-specific classifier to a gene-neutral classifier, such as "MET". Since "MET" was the only gene that had a highly frequent English sense, we could expect that the model trained on the other seven genes probably would not work very well on resolving its English meaning. The similar problems caused by the size of training set and skewed class distribution have been reported in ML research [25,26]. Nevertheless, the experiment of annotating a large set cancer trials demonstrated that the gene-neutral classifier would be very useful in this task. It is difficult to build a gene-specific classifier and optimize the parameters for every single gene, as the number of genes mentioned in cancer clinical trials keeps growing. The gene-neutral approach provides a scalable solution. Our study showed that the gene-neutral classifier could achieve reliable high performance for new trials, when enough samples were annotated. We expect that the performance of the gene-neutral classifier could be further improved, as more and more annotated samples are accumulated. Meanwhile, annotation time will be dramatically reduced as the system's predictions become more accurate.

In the future, we will further improve the classification performance by investigating other machine learning algorithms or ensembles of classifiers. We will integrate the gene-neutral approach into the workflow of cancer trial annotation. In addition, we will integrate methods developed here with the search function at MyCancerGenome.org, and measure its practical uses in information retrieval tasks of clinical trials.

## Conclusions

In this study, we developed a two-stage classifier to identify gene entities and the statuses of genetic lesions from clinical trial documents. Our system achieved an average accuracy of 83.7% when developed and tested on individually annotated genes. In addition, we conducted experiments and demonstrated the feasibility of building a gene-neutral classification approach for this task. To our best knowledge, this is one of the first attempts to accurately identify genetic information in clinical trial documents. We plan to apply these methods to facilitate information retrieval of gene-associated clinical trials.

## Competing interests

The authors declare that they have no competing interests.

## Authors' contributions

YW, MAL, and HX were responsible for the overall design, development, and evaluation of this study. CMM and PY developed the annotation guidelines and annotated the data set used for this study. BT worked with YW on the algorithm development. MJC and SMC provided the original data sets for this study. YW, MAL, and HX did the bulk of the writing, and CMM also contributed to writing and editing of this manuscript. All authors reviewed the manuscript critically for scientific content, and all authors gave final approval of the manuscript for publication.
